# Balloon Valvuloplasty in Congenital Critical Aortic Valve Stenosis in Neonates and Infants: A Rescue Procedure for the Left Ventricle

**DOI:** 10.3390/jcdd11050156

**Published:** 2024-05-18

**Authors:** Jochen Pfeifer, Axel Rentzsch, Martin Poryo, Hashim Abdul-Khaliq

**Affiliations:** Department of Pediatric Cardiology, Saarland University Medical Center, 66421 Homburg, Germany

**Keywords:** critical aortic valve stenosis, transcatheter balloon valvuloplasty, retrieval loop, neonate, cardiogenic shock, left ventricular dysfunction

## Abstract

Congenital critical aortic valve stenosis (CAVS) is a life-threatening disease requiring urgent treatment. First-line therapy is still controversial. The aim of our study was (1) to analyze retrospectively the patients of our institution who underwent balloon aortic valvuloplasty (BAV) due to CAVS and (2) describe the techniques for improved feasibility of intervention using microcatheters and retrieval loops. Twelve patients underwent 23 BAVs: 1 BAV was performed in 3 patients, 2 BAVs were performed in 7 patients, and 3 BAVs were performed in 2 patients. The peak trans-valvular pressure gradient (Δp) and left ventricular shortening fraction (LVSF) improved significantly in the first two interventions. In the first BAV, Δp decreased from 73.7 ± 34.5 mmHg to 39.8 ± 11.9 mmHg (*p* = 0.003), and the LVSF improved from 22.3 ± 13.5% to 31.6 ± 10.2% (*p* = 0.001). In the second BAV, Δp decreased from 73.2 ± 33.3 mmHg to 35.0 ± 20.2 mmHg (*p* < 0.001), and the LVSF increased from 26.7 ± 9.6% to 33.3 ± 7.4% (*p* = 0.004). Cardiac surgery during the neonatal period was avoided for all children. The median time to valve surgery was 5.75 years. Few complications occurred, namely mild-to-moderate aortic regurgitation, one remediable air embolism, and one intimal injury to the ascending aorta. We conclude that BAV is a successful emergency treatment for CAVS, resulting in left ventricular relief, clinical stabilization, and a time gain until cardiac surgery.

## 1. Introduction

Aortic valve (AV) stenosis is a rare congenital cardiac malformation with a prevalence of 2.2–6% [1,2]. “Critical” aortic valve stenosis (CAVS) implies a severe impairment of the left ventricle (LV) due to a high-grade stenosis and pressure overload of the LV. Of note, there is no standardised definition for CAVS [3]. AV dysplasia and hypoplasia of the aortic annulus are the main causes. In most cases, the AV has a unicuspid or bicuspid morphology [2,4] due to congenital commissural fusions. Additional cardiac malformations may exist, such as aortic hypoplasia or coarctation of the aorta.

CAVS results in low-output heart failure due to LV pressure overload with severely reduced LV systolic function, LV enlargement and eccentric dilatation, and mitral regurgitation. Endocardial fibroelastosis of the LV may occur as well [5]. Cardiomegaly and fetal hydrops may develop antenatally [6].

However, clinical manifestations vary from mild symptoms to cardiogenic shock after birth. Floppiness, pallor, nutritional disturbance, and failure to thrive are common initial symptoms. A typical systolic murmur may be missing, especially in cases of high-grade stenosis with low cardiac output.

Frequently, a definitive diagnosis is only made after clinical manifestation in the neonate or infant, as the rate of prenatally diagnosed CAVS by fetal ultrasound is still low [7]. In case of (imminent) cardiogenic shock, intravenous prostaglandin application should be started immediately after birth post diagnosis in order to maintain a sufficient systemic perfusion via a patent ductus arteriosus. Inotropic medication, mechanical ventilation, and balloon atrioseptostomy may also be indicated [8,9].

The further therapeutic approach can be challenging. It should always be aimed at LV pressure relief by enlargement of the aortic valvular orifice. It is still controversially discussed which technique—transcatheter balloon aortic valvulotomy (BAV) or cardiac surgery—is more beneficial [3,10,11,12,13,14,15,16]. Transcatheter BAV, first described by Labadibi et al. [17], has become the standard of care in most centers. The intention is to quickly reduce the stenosis and thus achieve improvement in LV function while avoiding open-heart surgery and cardio-pulmonary bypass in a critically ill neonate. Both retrograde and antegrade trans-valvular access are performed, depending on the feasibility of probing the stenotic valve.

The aim of this retrospective study was to analyze patients’ characteristics and the procedural parameters, effectiveness, complications, and outcomes of transcatheter BAV in CAVS in our institution. Stable support for guidewires and valvuloplasty catheters is particularly challenging in CAVS due to the valvular dysplasia and the small anatomical features in the relevant age group. In this regard, we also present rarely described techniques for peri-interventional wire and catheter support [18].

## 2. Material and Methods

After approval from the local ethics committee of Saarbruecken, Saarland, Germany (file number: 37/24), this single-center retrospective observational cohort study was performed at the Saarland University Medical Center in Homburg/Saar, Germany.

We retrospectively analyzed all patients with CAVS who underwent emergency transcatheter BAV as first-line therapy in our institution from January 2006 to December 2023. Primary surgical approach was not performed on any patient with CAVS. The criteria for the diagnosis of CAVS were depressed systolic LV function or clinical symptoms of heart failure or cardiogenic shock. Patients with associated cardiac malformations were excluded (e.g., hypoplastic left heart syndrome, Shone’s complex, ventricular septal defect, and others) but not those with extra cardiac malformations (e.g., aortic coarctation). We reviewed the relevant patients’ files as well as the echocardiographic and angiographic records in order to obtain the following data:

- Patients’ characteristics (sex, body weight, and age at intervention);
- AV cuspidity and diameter of the AV annulus;
- Balloon diameter and balloon/annulus ratio;
- Direction of the AV access (i.e., antegrade or retrograde);
- Left ventricular end-diastolic diameter (LVEDD (mm)) before BAV;
- Left ventricular shortening fraction (LVSF (%)) before and after BAV;
- Peak trans-valvular systolic pressure gradient (mmHg) before and after BAV;
- Post-interventional aortic regurgitation and other BAV-related complications (cardiovascular injuries, thrombosis, embolism, hemorrhage, and mortality);
- Time to AV surgery.

The Z scores were calculated according to the work of Cantenotti et al. (2017) [19] for the AV annulus and according to the work of Cantenotti et al. (2014) [20] for the LVEDD. A unicuspid (or unicommissural) aortic valve was assumed if the echocardiographic criteria for its diagnosis were met according to the work of Ewen et al. (2019) [21]: a single commissural attachment, rounded opposite margin, and eccentric valvular orifice. In bicuspid valves, there are two commissural attachment zones and one fused commissure with a reduced commissural height.

### 2.1. Statistics

The reduction of the trans-valvular pressure gradient as well as the improvement in the LVSF by BAV was evaluated using paired t-tests. The normality of the data was assessed by a Shapiro–Wilk test. A Kaplan–Meier analysis (time-to-event analysis) was performed to calculate the time to valvular surgery after BAV. IBM^©^ SPSS 28.0 statistics software and Microsoft^©^ Excel 2016 were used for the statistical analyses.

### 2.2. Percutaneous Catheterization

**Figure 1:**
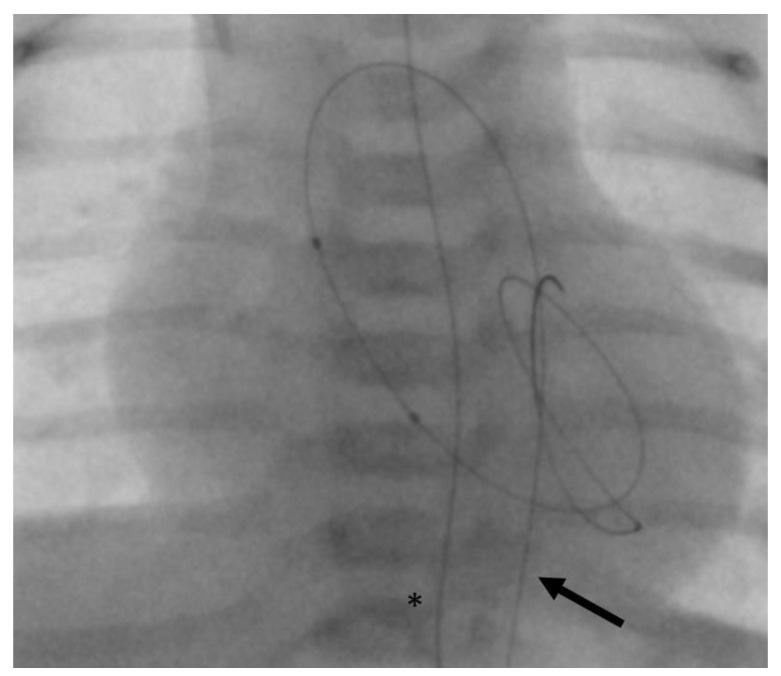
Angiography of balloon aortic valvulotomy (BAV) with retrograde access (posterior-anterior projection). A trans-aortic guidewire (arrow) is inserted deep into the left ventricle to ensure stable balloon support during BAV. The asterisk marks a gastric tube.

Femoral venous and arterial access was chosen in all patients. Four French introducer sheaths and 4 French angiographic catheters (Multipurpose A SPECIAL, Cordis^©^, Miami Lakes, FL, USA) were used. First, we aimed at trans-arterial retrograde probing of the AV using a coronary guidewire. If this was successful, then it may still have been difficult to pass through the severely stenotic AV orifice with the angiographic catheter. In these cases, a 2.7 French microcatheter (Rebar™ 18, ev3 Neurovascular, Irvine, CA, USA) was introduced beforehand in order to provide more support for the angiographic catheter and thus to allow its trans-valvular passage. For retrograde BAV, the guidewire usually can be positioned stably within the LV (Figure 1).

**Figure 2:**
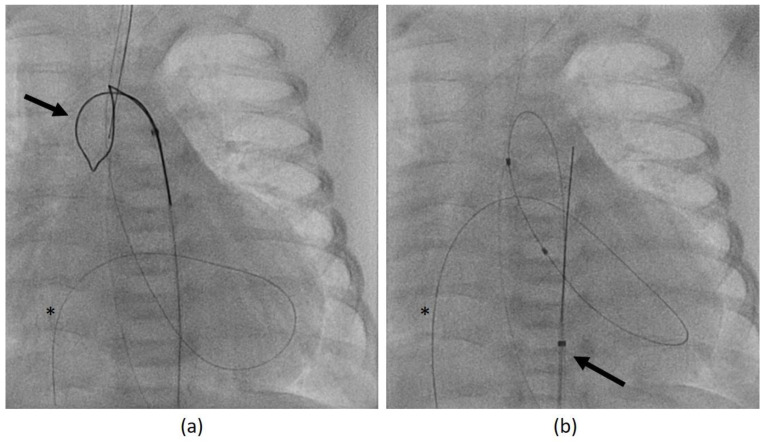
Angiography of balloon aortic valvulotomy (BAV) with antegrade access (posterior-anterior projection). (**a**) A coronary guidewire (asterisk) was positioned trans-venously up to the ascending aorta, where it was snared by a trans-aortic inserted loop (arrow). (**b**) After catching of the guidewire (asterisk), it was attached by the snare catheter (arrow) in the descending aorta to ensure stable balloon support during BAV.

If retrograde catheterization was not feasible, then a trans-venous antegrade approach was accomplished via inferior the vena cava, right atrium, foramen ovale, left atrium, and LV. After passing the AV into the ascending aorta, the guidewire was caught using a retrieval loop (Multi-Snare^©^, pfm medical mepro gmbh, Nonnweiler-Otzenhausen, Germany; 5 or 10 mm in diameter) which was inserted retrogradely into the aorta (Figure 2a). Then, it was possible to pull the wire into the descending aorta and fixate it stably during the BAV (Figure 2b).

Laevocardiography was conducted in order to localize and measure the diameter of the aortic annulus in all cases. For aortic valvuloplasty, we used either coronary balloon catheters or valvuloplasty catheters (Tyshak mini^©^, NuMED, Inc., Hopkinton, NY, USA) which were introduced over the guidewire. BAV was conducted stepwise with gradually increasing balloon diameters. We administered Heparin (100 IU per kg body weight) and antibiotic prophylaxis during the procedure.

## 3. Results

### 3.1. Patients’ Characteristics, Cardiac Parameters, and Procedural Parameters

For underlying CAVS, BAV was performed in 12 patients (83.3% male), of which 9 patients underwent a second procedure and 2 patients underwent a third procedure. In total, 23 BAV procedures were performed. A renewed increase in the pressure gradient or worsening of the left ventricular systolic function were indications for re-intervention. One patient had an additional aortic coarctation. Diagnoses were made via fetal ultrasound in 2/12 patients (16.7%) and postnatal echocardiography in 10/12 (83.3%) due to a systolic murmur or symptoms.

A unicuspid AV was present in 9/12 patients (75%), and a bicuspid AV was in 3/12 (25%). In the latter, the right and the left coronary cusps were fused in two cases, whereas the right coronary and the non-coronary cusps were fused in one case. At the time of the first BAV, the median Z score for the AV annulus was −1.25 (range: from −4.1 to +2.8), and the median Z score for LVEDD was +1.8 (range: from −2.0 to +6.2). Retrograde BAV could be achieved in 6/12 (50%) patients in the first intervention, in 6/9 (66.7%) patients in the second, and 2/2 (100%) patients in the third BAV. Antegrade BAV was performed for all other procedures. The balloon/AV annulus ratio for the first BAV was at a median of 0.90 (range: 0.7–1.1), a median of 1.0 for the second BAV (range: 0.84–1.16), and a median of 1.23 in both interventions for the third BAV.

**Figure 3:**
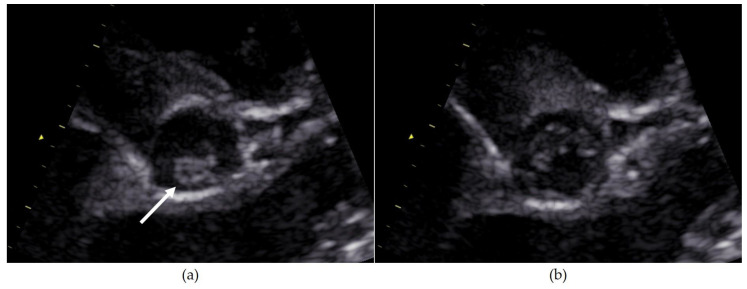
Echocardiographic images of a unicuspid valve. (**a**) Single commissural attachment zone (arrow). (**b**) Eccentric, “pinhole-like” valvular opening during systole. The margin opposite the commissural attachment is rounded.

The patients’ characteristics and morphological and functional cardiac parameters, as well as the procedural parameters, are summarized in Table 1, and their case-based descriptions are in Table 2. Figure 3 shows illustratively an echocardiographic image of a unicuspid valve (case 3).

### 3.2. Effectiveness of BAV

#### 3.2.1. First BAV

**Figure 4:**
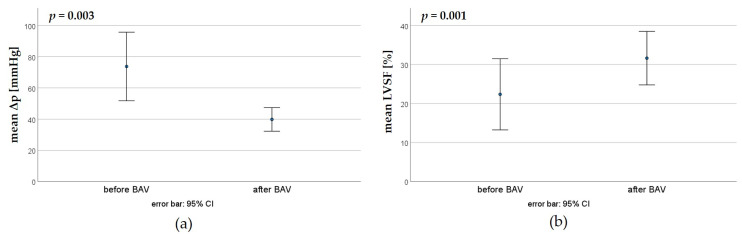
Effectiveness of the first BAV; paired *t*-tests. (**a**) Reduction in the trans-valvular pressure gradient (Δp). (**b**) Increase in the left ventricular shortening fraction (LVSF) by the first BAV.

In the first BAV, the peak pressure gradient could be reduced from a mean of 73.7 ± 34.5 mmHg to 39.8 ± 11.9 mmHg, whereas the LVSF increased from a mean of 22.3 ± 13.5% to 31.6 ± 10.2% (Figure 4).

#### 3.2.2. Second BAV

**Figure 5:**
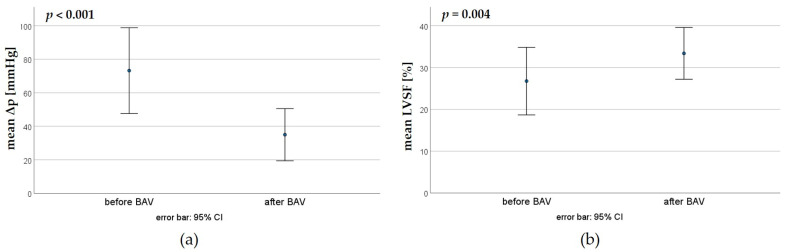
Effectiveness of the second BAV; paired *t*-tests. (**a**) Reduction in the trans-valvular pressure gradient (Δp). (**b**) Increase in the left ventricular shortening fraction (LVSF) by the first BAV.

Nine patients underwent a second BAV. Here, the peak pressure gradient could be reduced from a mean of 73.2 ± 33.3 mmHg to 35.0 ± 20.2 mmHg, whereas the LVSF increased from a mean of 26.7 ± 9.6% to 33.3 ± 7.4% (Figure 5).

#### 3.2.3. Third BAV

**Figure 6:**
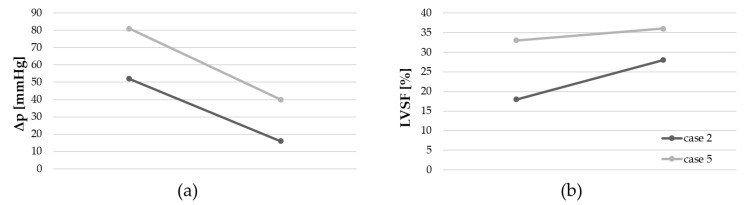
Third BAV. (**a**) Reduction in the trans-valvular pressure gradient. (**b**) Increase in the left ventricular shortening fraction (LVSF).

A third BAV was performed in only two patients (cases 2 and 5, Table 2). A reduction in the pressure gradient and an increase in the LVSF were achieved in both interventions (Figure 6).

### 3.3. Complications

#### 3.3.1. Aortic Regurgitation

**Figure 7:**
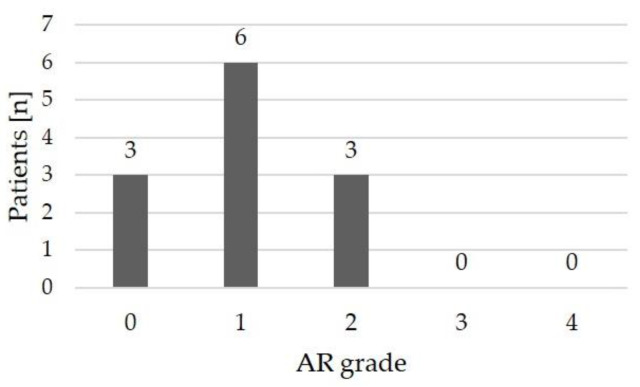
Grading of the aortic regurgitation (AR) immediately following balloon aortic valvulotomy.

Echocardiographic assessment following the most recent BAV revealed no or only mild aortic regurgitation in 9/12 patients (75%), while 3/12 patients (25%) had moderate aortic regurgitation (Table 2, Figure 7). High-grade regurgitation did not occur.

#### 3.3.2. Miscellaneous Complications

**Figure 8:**
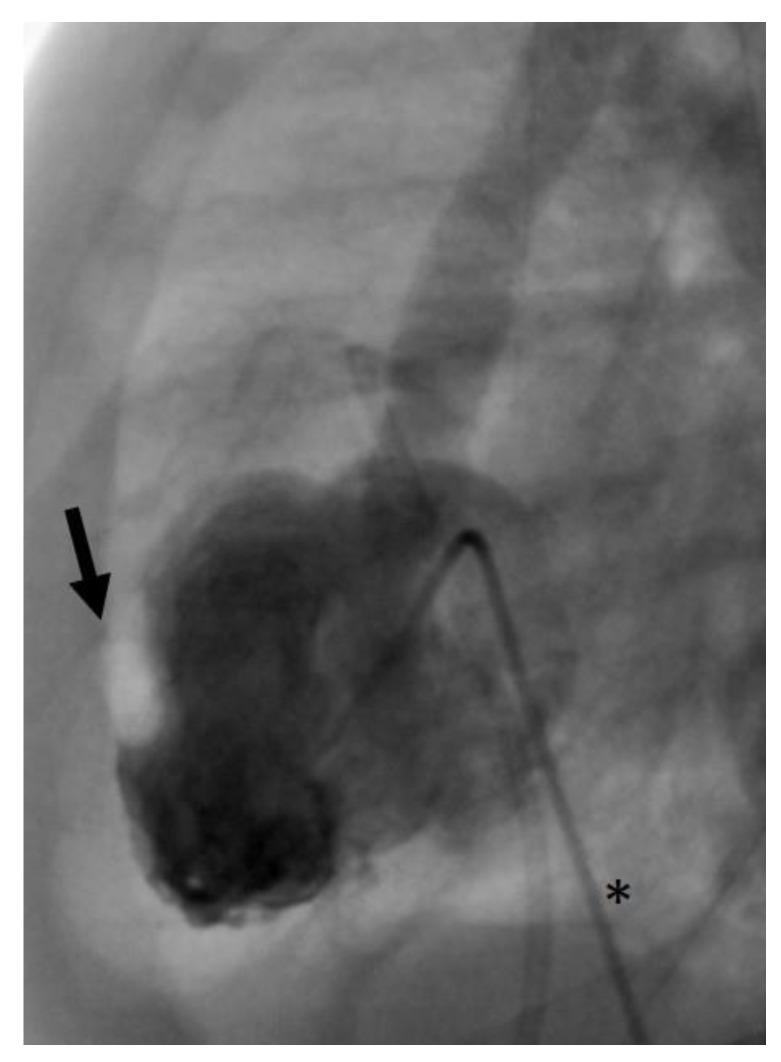
Angiographic image, lateral view. Air bubble (arrow) ventrally in the left ventricle during catheterization. The asterisk marks an angiographic catheter.

**Figure 9:**
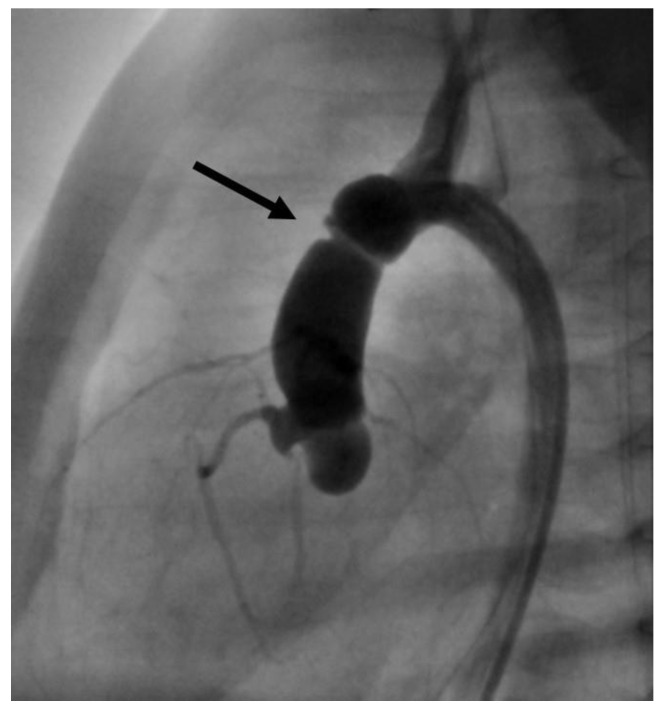
Angiographical image, lateral view. Intimal lesion with consecutive constriction in the ascending aorta (arrow).

- In case 2, there was an air embolism in the left ventricle during the patient’s first catheterization. Due to depressed LV function with low contractility, the air bubble remained ventrally in the LV (Figure 8), where we managed to extract it with the catheter.

- In the same patient, an aortic wall injury resulting in a constriction of the intima following the second BAV occurred (Figure 9). However, this constriction was without significant stenosis and was corrected at the time of necessary aortic valve repair.

- Further complications did not occur, and there was no mortality.

### 3.4. Follow-Up

**Figure 10:**
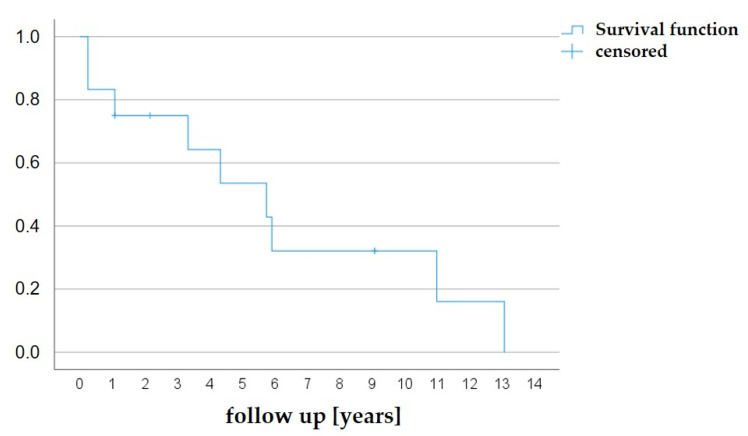
Kaplan–Meier analysis. Freedom from AV surgery after BAV. The Kaplan–Meier estimator was 5.75 years.

The follow-up duration was a median of 8.96 years (1.0–19.9). No patient was lost to follow-ups. To date, 9/12 patients (75.0%) had to undergo a surgical AV reconstruction due to progressive aortic regurgitation, aortic re-stenosis, or combined stenosis and regurgitation. Thereof, n = 6 patients had a unicuspid valve procedure, and n = 3 had a bicuspid valve procedure. For all cases, valvular cuspidity was confirmed by the surgeon. The earliest time for an operation was after 3 months. The median time to surgery (Kaplan–Meier estimator) was 5.75 years (Figure 10).

## 4. Discussion

In the case of CAVS, high-grade LV dysfunction with diminished contractility and resultant low cardiac output usually leads to rapid clinical deterioration until cardiogenic shock of the neonate [9,22]. Therefore, urgent intervention is indicated. Regarding the further prognosis, saving the depressed LV has the topmost priority. Both open-heart surgery and transcatheter BAV are possible approaches for initial treatment [10]. Nevertheless, the optimal first-line therapy is still controversial [3]. In our center, a transcatheter intervention is performed as a first-line treatment in all cases of CAVS in neonates and infants. Primary aortic valve surgery is usually conducted in older children who undergo elective valvular reconstruction, the Ross procedure, or valve prosthesis.

In the present study, we analyzed a case series of 12 children with CAVS who underwent BAV. In all patients, aortic valve dysplasia in terms of either unicuspid (75%) or bicuspid (25%) AV was diagnosed. Although there is rather low incidence (0.02%) for unicuspid aortic valves in the overall population [21,23], studies suggest that it is common in severe congenital aortic valve stenosis. Nevertheless, the data on its incidence varied greatly in previous publications. In a recent study involving a cohort of 16 neonates with aortic valve stenosis and LV dysfunction, the distribution of valve morphologies was 68.75% unicuspid, 25% bicuspid, and 6.25% tricuspid, according to the authors [4]. Matsushima et al. stated that “almost all” stenotic aortic valves “requiring intervention in childhood” are due to an underlying unicuspid morphology [24]. In contrast, in the study by Zain et al. on 25 newborns with CAVS, most were diagnosed with a bicuspid morphology (72%), followed by tricuspid morphology (24%) and unicuspid at only 4% [10]. The reason for these differences probably lies in the still inconsistent anatomical assessment and nomenclature of aortic valve morphology.

Bicuspid AVs are present in 1–2% of the population [23]. Fusion of the right and left coronary cusps is the most common phenotype (70–80%), followed by fusion of the right coronary and non-coronary cusps (20–30%) and fusion of the left coronary and non-coronary cusps (3–6%), according to Michelena et al. [25]. In our cohort, we found the first type in two patients, the second in one patient, and the third in none.

At the time of the first BAV, the median age was 3 days. Only in 2/12 patients was a CAVS diagnosis made with fetal sonography. This is consistent with the results of Freud et al. [7], who also found a low rate (8.5%) of prenatally diagnosed CAVS. Earlier detection would be desirable in order to subject the neonates to immediate intervention. In a recent study, Sylwestrzak et al. demonstrated that the best outcome is achieved if CAVS is diagnosed prenatally, and postnatal BAV is performed within the first few days of life [26].

Nine of our patients (75.0%) underwent a second BAV, and two patients (16.7%) needed a third one. Previous studies also described a frequent need for re-intervention after a successful first BAV, especially among younger children [13,27]. In our patients, both significant improvement in LV function (i.e., LV shortening fraction) and significant reduction of the peak trans-aortic pressure gradient could immediately be achieved. The end-diastolic LV diameter usually normalizes within only several months to years due to pressure relief. A recurrent LV dilatation may be an indication of relevant aortic regurgitation [13].

Petit et al. suggested that BAV is particularly successful in cases of high-grade stenosis due to small valve orifice areas and unicuspid AVs [28], which is typical for CAVS. Repeated BAV may be required because AV stenosis tends to recur following the initial BAV due to the recovery of LV function and consecutively increasing LV output. The development of scar tissue may promote re-stenosis as well. Moreover, it is advisable to start with low balloon/annulus ratios using coronary balloon catheters to prevent AV injury and high-grade aortic regurgitation. The balloon diameters can then be increased gradually in order to achieve a stepwise enlargement of the AV orifice area. The usually recommended balloon/annulus ratios are 0.7–1.0. Nevertheless, higher ratios might be required for an effective dilatation due to the rigidity of severely dysplastic AVs [8,10,14,29]. In our analysis, the balloon/annulus ratios were from 0.9 in the first BAV up to a maximum of 1.23 in the third BAV. In light of the early re-intervention within days in our patients, it seems sensible to already consider higher ratios for the first BAV.

By applying an interventional method as a rescue procedure, cardiac surgery, including a cardio-pulmonary bypass, can be avoided in neonates, who are a particularly vulnerable cohort with high perioperative mortality [30], which is even higher in cases of cardiogenic shock. In our study, the estimated time to AV surgery was 5.75 years. Among others, severe left ventricular dysfunction is a risk factor for AV surgery following neonatal BAV, according to Kido et al. [31]. However, the time gained until the first surgery is worthwhile because patients with congenital AV stenosis are exposed to a high mortality risk for multiple AV operations, including AV reconstruction, the Ross procedure, or AV prosthesis [32,33,34,35,36]. Thus, future perioperative risks due to typical intrathoracic adhesions [37,38] might be reduced by postponing surgery to later childhood. On the other hand, there are data that valve replacement cannot be postponed by BAV [39]. Moreover, some authors generally performed primary cardiac surgery [40] because it offers a precise AV commissurotomy and reconstruction, in contrast to arbitrary valve dilatation [11], and longer freedom of re-intervention [41]. Hill et al. described no differences in long-term outcomes for either primary BAV or a surgical approach but a higher rate of re-intervention after BAV [15]. However, both are palliative approaches, and AV surgery will be necessary in most cases anyway. Our data support the hypothesis of rescue interventional procedures to improve survival in critically ill neonates with primarily diminished LV contractility.

When performing BAV, probing highly restrictive AVs with a catheter is often challenging. We describe a technique to provide more support for the angiographic catheter by inserting it via a guide wire and an additional microcatheter. In principle, either trans-arterial retrograde or trans-venous antegrade catheterization is possible. If a retrograde approach is unfeasible due to severely dysplastic and stenotic aortic valves featuring tiny orifice areas as well as systolic counter flow, then trans-venous antegrade catheterization should be considered, which provides equally effective results [42]. Here, stable and safe positioning of the balloon during BAV can be technically delicate. For this purpose, it is useful to fixate the guiding wire in the descending aorta using a retrieval loop, as first described by Rao et al. [18] in a trans-umbilical approach. Other options for intra-procedural balloon stabilization are rapid pacing or inducing short-term asystole by adenosine administration [43]. In three of our patients, antegrade access had to be chosen in the first intervention, but retrograde access was feasible in the second (n = 1) or third (n = 2) procedure. This can be explained by a successfully increased valvular orifice area from the initial dilatation, which thereafter facilitated catheter passage in the following interventions.

The antegrade approach is associated with mitral valve injury, whereas there is a risk of arterial thrombosis in retrograde catheterization [42,44]. Neither occurred in our population. In our study, few BAV-associated complications occurred. The greatest risk exists for the development of aortic regurgitation, especially for repeated BAV [27]. In 75.0% of our patients, no or mild aortic regurgitation was detectable, and in 25.0%, moderate regurgitation was found. There was no case of severe aortic valve insufficiency, which usually only increases over time according to Rao [45]. Another adverse event was an air embolism in the LV. Due to poor LV function with significantly reduced contractility, the air remained in the LV without embolization into systemic arteries. Therefore, the bubble could be aspirated by a catheter. The most remarkable complication was an intimal injury to the distal ascending aorta, which was also described as typical in a study by Brown et al. [46]. In our case, the lesion was electively corrected in combination with the surgical AV reconstruction. There was no peri-interventional mortality, nor was there mortality in the follow-ups in our study.

The limitations of our study are the retrospective approach as well as the limited number of patients, which was based on both the low incidence of CAVS and the single-center analysis. Due to the small number of patients, predictors for the future need re-intervention, which can hardly be provided.

## 5. Conclusions

In critical aortic valve stenosis, balloon valvuloplasty is a successful primary treatment resulting in prompt left ventricular relief and stabilization of critically ill neonates. Ideally, this means that cardiac surgery can be avoided during the neonatal period and postponed until later in childhood. No mortality was observed in our cohort. Thus, the transcatheter approach represents an effective emergency yet palliative rescue procedure for the LV with an acceptably low rate of complications. Nevertheless, it seems advisable to choose first-line therapy according to the experience of the respective treatment team.

## Figures and Tables

**Table 1:** Overall patients’ characteristics, AV cuspidity, and access direction.

Variable	First Valvuloplasty *n* = 12	Second Valvuloplasty *n* = 9	Third Valvuloplasty *n* = 2
Age at procedure: days (range)	3 (1–55)	34 (4–83)	49 and 104
Female: *n* (%)	2 (16.7)	1 (11.1)	0
Male: *n* (%)	10 (83.3)	8 (88.9)	2 (100)
Body weight: kg (range)	3.2 (2.6–5.1)	3.9 (2.7–5.1)	3.4 and 5.7
Unicuspid AV: *n* (%)	9 (75.0)	8 (88.9)	2 (100)
Bicuspid AV: *n* (%)	3 (25.0)	1 (11.1)	0
Antegrade BAV: *n* (%)	6 (50.0)	3 (32.3)	0
Retrograde BAV: *n* (%)	6 (50.0)	6 (66.7)	2 (100)

Data are illustrated as absolute numbers and percentages, with median and range. AV = aortic valve; BAV = balloon aortic valvuloplasty.

**Table 2:** Case-based description of cardiac and procedural parameters.

Case	Sex	Valve Cuspidity	Number of Interventions	Age (d)	Weight (kg)	LVEDD (mm) (Z Score)	AV Annulus (mm) (Z score)	Maximum Balloon Diameter (mm)	Balloon/Annulus Ratio	Antegrade (A) or Retrograde (R) BAV	LVSF before BAV (%)	LVSF following BAV (%)	Maximal Δp before BAV (mmHg)	Maximal Δp following BAV (mmHg)	AR following BAV (Grade 0–4)
1	m	UVC	1/2	3	3.6	30 (+5.4)	6.0 (−2.3)	5.5	0.91	R	13	23	37	20	0
			2/2	7	3.6	30 (+5.4)	6.0 (−2.3)	7.0	1.16	R	22	31	42	18	1
2	m	UCV	1/3	1	2.7	25 (+3.7)	5.5 (−2.4)	4.5	0.81	A	12	23	50	50	0
			2/3	4	2.7	26 (+4.2)	5.5 (−2.4)	6.0	1.09	A	21	30	60	36	1
			3/3	49	3.4	25 (+3.0)	6.5 (−1.5)	8.0	1.23	R	18	28	52	16	2
3	m	UCV	1/2	55	5.1	35 (+6.2)	9.0 (+0.9)	7.0	0.77	R	4	20	60	45	0
			2/2	70	5.1	34 (+5.8)	9.0 (+0.8)	8.0	0.88	R	15	23	50	21	1
4	m	UCV	1/2	29	3.9	25 (+2.8)	9.5 (+2.5)	7.0	0.73	R	15	27	90	50	1
			2/2	34	3.9	25 (+2.8)	9.5 (+2.5)	8.0	0.84	R	20	34	70	25	1
5	m	UCV	1/3	6	3.0	21 (+1.4)	6.0 (−1.9)	6.0	1.00	A	11	n.a.	55	36	1
			2/3	9	2.8	21 (+1.5)	6.0 (−1.7)	7.0	1.16	A	n.a.	n.a.	62	30	1
			3/3	104	5.7	19 (−1.4)	6.5 (−3.1)	8.0	1.23	R	33	36	81	40	1
6	m	UCV	1/2	1	3.4	21 (+1.3)	7.5 (+0.5)	7.0	0.93	R	44	50	130	50	0
			2/2	21	3.7	21 (+1.1)	7.5 (+0.2)	7.0	0.93	R	45	48	50	15	0
7	m	UCV	1/2	32	3.5	24 (+2.6)	7.0 (−0.6)	6.0	0.85	A	25	25	104	50	0
			2/2	83	4.9	24 (+0.5)	8.0 (−1.5)	8.0	1.00	R	33	39	130	40	2
8	f	BCV	1/1	7	2.9	21 (+1.6)	5.5 (−2.6)	5.0	0.90	R	41	43	85	45	2
9	f	UCV	1/2	2	3.2	15 (−2.0)	5.0 (−4.0)	5.0	1.00	A	38	37	50	30	0
			2/2	78	4.1	17 (−1.6)	5.5 (−3.9)	5.0	0.90	A	33	32	65	50	0
10	m	BCV	1/1	3	2.6	20 (+1.4)	8.5 (+2.8)	6.0	0.70	A	25	44	125	25	1
11	m	BCV	1/2	2	2.6	21 (+2.0)	6.5 (−0.1)	6.0	0.92	R	20	31	80	52	0
			2/2	55	4.6	28 (+3.7)	7.5 (−0.7	8.0	1.06	R	25	30	130	80	0
12	m	UCV	1/1	2	3.2	21 (+1.3)	5.0 (−4.1)	5.5	1.10	A	9	25	19	25	1

Data are illustrated as absolute numbers and percentages. A = antegrade; AR = aortic regurgitation; AV = aortic valve; BCV = bicuspid aortic valve; LVEDD = left ventricular end-diastolic diameter; LVSF = left ventricular shortening fraction; R = retrograde; UCV = unicuspid aortic valve; Δp = peak trans-valvular pressure gradient; n.a. = not applicable due to left ventricular dyssynchrony.

## Data Availability

The data underlying the present study are available on request (corresponding author).
